# Cancer-associated fibroblast-derived Dickkopf-1 suppresses NK cell cytotoxicity in breast cancer

**DOI:** 10.21203/rs.3.rs-4202878/v1

**Published:** 2024-04-08

**Authors:** Roberta Faccio, Seunghyun Lee, Biancamaria Ricci, Jennifer Tran, Jiayu Ye, David Clever, Emily Eul, Julia Wang, Pamela Wong, Cynthia Ma, Todd Fehniger

**Affiliations:** Washington University in St. Louis; Washington University in St. Louis; Washington University in St. Louis; Washington University in St. Louis; Washington University in St. Louis; Washington University in St. Louis; Washington University in St. Louis; Washington University in St. Louis; Washington University in St. Louis; Washington University in St. Louis; Washington University in St. Louis

## Abstract

Breast cancer is poorly immunogenic, hence able to evade T cell recognition and respond poorly to immune checkpoint blockade. Breast cancer cells can also evade NK cell-mediated immune surveillance, but the mechanism remains enigmatic. Dickkopf-1 (DKK1) is a Wnt/b-catenin inhibitor, whose levels are increased in breast cancer patients and correlate with reduced overall survival. DKK1 is expressed by cancer-associated fibroblasts (CAFs) in orthotopic breast tumors and patient samples, and at higher levels by bone cells. While bone-derived DKK1 contributes to the systemic elevation of DKK1 in tumor-bearing mice, CAFs represent the primary source of DKK1 at the tumor site. Systemic or bone-specific DKK1 targeting reduces primary tumor growth. Intriguingly, specific deletion of CAF-derived DKK1 also limits breast cancer progression, regardless of its elevated levels in circulation and in the bone. DKK1 does not support tumor proliferation directly but rather suppresses the activation and tumoricidal activity of NK cells. Importantly, increased DKK1 levels and reduced number of cytotoxic NK cells are detected in breast cancer patients with progressive bone metastases compared to those with stable disease. Our findings indicate that DKK1 creates a tumor-supporting environment through the suppression of NK cells in breast cancer.

## Introduction

Breast cancer is one of the most frequently diagnosed malignancies in women worldwide^1^. Despite survival benefits if patients are diagnosed early, approximately 20–30% of breast cancer survivors experience metastatic relapse within 5–10 years of curative-intent therapy. Therapeutic options available for metastatic breast cancer are largely palliative and majority of these patients would succumb to the disease^2^. Thus, it is very important to find long term and highly effective therapeutic approaches to prevent recurrence.

Immune checkpoint blockade (ICB) has been FDA approved to treat a variety of cancers previously considered uncurable. For breast cancer, anti-PD-1 mAb in combination with chemotherapy is now a standard-of-care for early (Keynote-522) and advanced (Keynote-355) triple-negative breast cancer (TNBC)^3, 4^. However, over 30% of TNBC patients do not benefit from ICB, and ICB is also not effective for the more common ER^+^ breast cancer^5^. Thus, combining ICB with other conventional therapies remains an active area of investigation in breast cancer.

Key factors responsible for the poor therapeutic responses to ICB in breast cancer include low tumor mutational burden, resulting in fewer neo-antigens and less T cell recognition^6^, recruitment of suppressive immune cells, and exclusion of lymphocytes at the tumor sites^7^. Recent analysis of circulating immune cell populations in breast cancer patients refractory to neoadjuvant chemotherapy revealed an apparent increase in the proportion of dysfunctional CD56^dim^/CD16^−^ NK cells compared to patients achieving a pathological Complete Response (pCR)^8^. NK cells are innate immune populations offering a first line of defense against incipient tumors, including breast cancer^9^. NK cell intratumoral and peritumoral abundance correlate with an elevated pCR rate in breast cancer patients undergoing neoadjuvant chemotherapy^10^. Furthermore, preclinical evidence suggests that activated NK cells could potentiate response to ICB in MHC class I low tumors^11^. Thus, understanding the mechanisms that drive systemic immunosuppression, including NK cell inactivation, could potentially improve response to ICB in breast cancer patients.

Dickkopf-1 (DKK1) is a soluble inhibitor of the Wnt/b-catenin signaling pathway, primarily recognized for its role in bone homeostasis and cancer-induced osteolytic bone disease^12, 13^. Elevated levels of DKK1 in circulation and/or in tumor tissues also correlate with poor prognosis in numerous cancer types, regardless of the bone involvement^14, 15, 16, 17, 18, 19^. Direct effects of DKK1 on tumor cell Proliferation and survival have been described in head and neck cancer, pancreatic ductal adenocarcinoma, and esophageal squamous cell carcinoma^20, 21, 22^. Our lab and others have also demonstrated that DKK1 drives the accumulation of suppressive myeloid populations in melanoma, lung carcinoma, prostate cancer and gastric cancer^18, 23, 24, 25, 26, 27^, ultimately reducing anti-tumor T cell and NK cell responses. Thus, DKK1 targeting has been successfully used in combination with ICB to enhance anti-tumor immunity in mouse models of melanoma and gastric cancer^24, 26, 28^. In breast cancer, high levels of DKK1 have been associated with poor prognosis and metastatic dissemination to bone^15^. The pro-tumorigenic effects of DKK1 in breast cancer have been mainly attributed to its ability to increase bone resorption, thus creating a favorable environment for tumor dissemination to bone^15, 29^. However, whether DKK1 exerts systemic immune suppressive effects and/or generates a local immune suppressive environment at the primary tumor site has never been reported.

In this study, we demonstrate the direct inhibitory effects of DKK1 on NK cell cytotoxicity during breast cancer progression. We show that DKK1 is expressed by cancer-associated fibroblasts (CAFs) infiltrating primary tumors, and at higher levels by bone cells. While bone-derived DKK1 contributes to the systemic elevation of DKK1 during tumor progression, CAFs represent the primary source of DKK1 at the tumor site, suppressing NK cell-mediated tumoricidal activity. Importantly, increased DKK1 levels and a reduced number of cytotoxic NK cells are also detected in breast cancer patients with progressive disease. Our work positions DKK1 as a negative modulator of NK cell cytotoxicity and raises the importance of evaluating DKK1 levels before administration of NK-directed therapies.

## Results

### DKK1 augments breast cancer progression.

**Figure 1:**
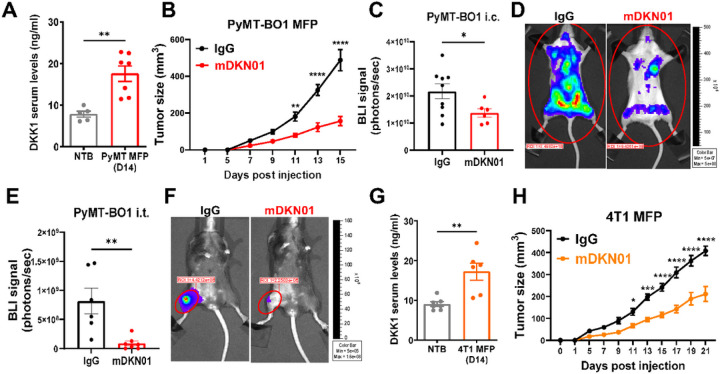
DKK1 augments breast cancer progression. (A) DKK1 serum levels were measured by ELISA in 6–8 weeks old female C57BL/6 WT mice with no tumors or 2 weeks after the inoculation of PyMT-BO1 breast cancer cells into the mammary fat pad (MFP). (B) Tumor growth in the MFP was determined by caliper measurements in WT mice inoculated with PyMT-BO1 (n=5 mice/group) receiving mDKN01 (10mg/kg) or control IgG antibody i.p. every other day. (C-F) Tumor progression was determined by BLI in mice inoculated with PyMT-BO1 cells (i.c.; 104 cells, albino C57BL/6, C, D) or intratibially (i.t.; 104 cells, C57BL/6, E, F) followed by administration of mDKN01 or control IgG antibody every other day. (G) DKK1 serum levels were measured by ELISA in 6–8 weeks old female BALB/c WT mice with no tumors or 2 weeks after the inoculation of 4T1 breast cancer cells into MFP. (H) Primary tumor growth was evaluated by caliper measurements in WT mice inoculated with 4T1 cells (n=4 mice/group) into the MFP receiving mDKN01 (10mg/kg) or control IgG antibody i.p. every other day. Results represent mean +/− SEM. Unpaired t-test (A, C, E, G), Two-way ANOVA followed by Bonferroni multiplecomparison test was used to determine significance (B, H). * P < 0.05, ** P < 0.01, *** P < 0.001 **** P < 0.0001.

To determine the role of DKK1 in breast cancer progression, we first measured DKK1 serum levels in C57BL/6 mice orthotopically injected with luminal B, ER^+^, hormone-resistant PyMT-BO1 cell line into the mammary fat pad (MFP). Serum DKK1 levels were significantly increased 14 days post inoculation (Fig. 1A), recapitulating the elevated levels observed in breast cancer patients^17^. Next, we administered the DKK1-neutralizing monoclonal antibody mDKN01 (10mg/kg), every other day following tumor inoculation and found a significant reduction in primary tumor growth compared to isotype control (IgG) (Fig. 1B). To further investigate the role of DKK1 during tumor dissemination, we injected the firefly luciferase-conjugated PyMT-BO1 cell line into albino C57BL/6 mice either intracardiacally (i.e.) or in the tibias (i.t.), followed by treatment with mDKN01. Bioluminescence imaging (BLI) showed significantly reduced tumor growth at all sites, including to visceral organs (Fig. 1C, D) and bone (Fig. 1E, F).

DKK1 levels were also significantly increased 14 days post inoculation in BALB/c mice orthotopically injected with the triple-negative 4T1 tumor line (Fig. 1G). Administration of mDKN01 (10mg/kg) led to a significant reduction in primary tumor growth compared to IgG control (Fig. 1H). Similarly, DKK1 neutralization significantly reduced the growth of the luminal B, ER/PR^+^, hormone-sensitive EO771 breast cancer cell line (Supp Fig. 1A). These results demonstrate the involvement of DKK1 in supporting tumor progression and the therapeutic benefit of a DKK1 targeting agent in various breast cancer subtypes.

### DKK1 is expressed in the tumor microenvironment and in bone.

**Figure 2:**
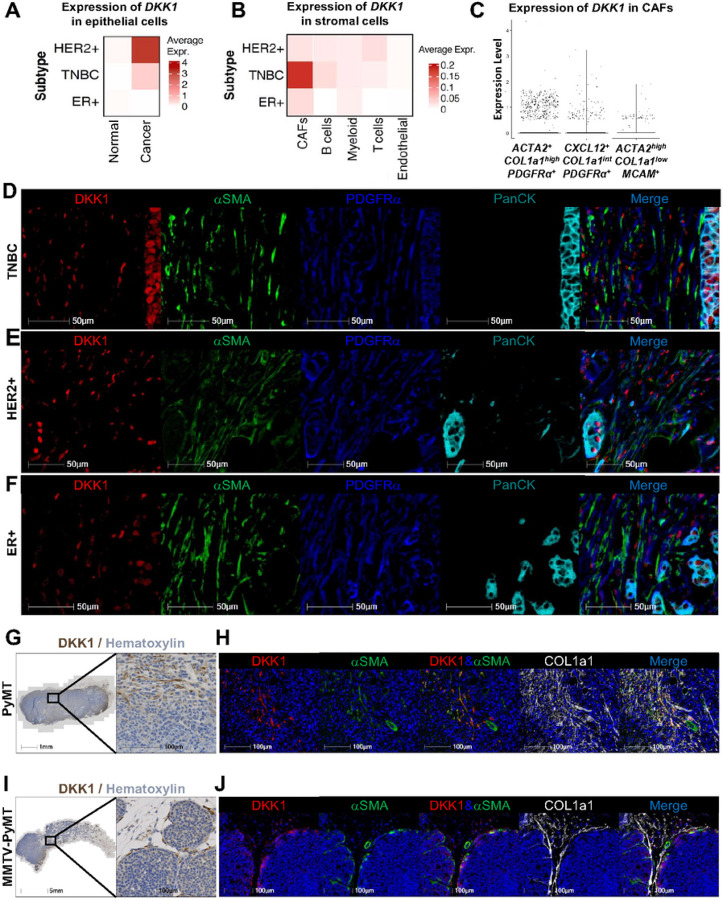
DKK1 is expressed by cancer-associated fibroblasts. (A) Heatmap visualization of DKK1 expression in normal and human breast cancer epithelial cells (GSE176078). (B) Heatmap visualization of DKK1 expression in the stromal compartment of HER2+, triple-negative, and ER+ human breast cancer subtypes. (C) Violin plot of DKK1 expression in CAF subsets defined by expression of indicated markers. (D-F) Multiplex immunohistochemistry (IHC) of triple-negative (D), HER2+ (E) and ER+ (F) human breast cancer subtypes stained for DKK1 (red), aSMA (green), PDGFRa (blue) and panCK (cyan). (G-J) DKK1 IHC (G, I) and multiplex IHC (H, J) of orthotopic PyMT and spontaneous MMTV-PyMT breast tumors stained for DKK1 (brown or red), aSMA (green), COL1a1 (white) and hematoxylin for nuclear visualization (blue).

In breast cancer, high levels of circulating DKK1 correlate with reduced survival and metastatic dissemination, especially to the bone^15, 17^. To assess expression of *DKK1* at tumor site, we analyzed a single-cell RNA seq dataset from 26 primary human breast tumors, including 11 ER^+^ (Luminal A or B), 5 HER2^+^, and 10 triple-negative breast cancers (TNBC) (GSE176078^30^). UMAP visualization resulted in 4 different clusters (endothelial cells, immune cells, epithelial cells, and cancer-associated fibroblasts (CAFs)), annotated by using canonical and signature-based markers^31^ (Supp Fig. 2A, B). *DKK1* was found to be mainly expressed in the cancer epithelial cells in the HER2^+^ subtype, and to a less extent in TNBC, while barely detectable in the normal epithelial cells (Fig. 2A). To our surprise, we also detected *DKK1* expression, albeit limited, in the stromal compartment, where *DKK1* was mainly detected within CAF clusters in TNBC, and to a lower extent also in the HER2^+^ and ER^+^ subtypes (Fig. 2B). Further subtype analysis based on previous breast cancer CAF classification^32, 33^ (Supp Fig. 2C, D), showed the highest *DKK1* expression in *ACTA2*^+^*COL1a1*^*high*^*PDGFRa*^+^ myofibroblasts (Fig. 2C). Based on these findings, we performed multiplex immunohistochemistry (IHC) using human breast cancer tissue microarrays containing TNBC, HER2^+^ and ER^+^ subtypes. DKK1 staining was observed in some cancer epithelial cells, identified by the co-expression with a pan-cytokeratin (PanCK) marker, and in PDGRFa^+^ and aSMA^+^ stromal populations in all tumor types (Fig. 2D–F), while it was not detected in the terminal duct lobular unit of normal breast tissue (Supp Fig. 2E).

Next, we investigated DKK1 expression in the PyMT, 4T1, and EO771 tumor models. While *Dkk1* was barely detectable in the tumor cell lines *in vitro* (Supp Fig. 1D), *Dkk1* transcripts were detected in the tumor mass 14 days post-inoculation (Supp Fig. 1B, E). Similar to the patient samples, IHC showed DKK1 staining in stromal cells with an elongated, fibroblast-like morphology in the PyMT orthotopic tumors (Fig. 2G) and in the spontaneous MMTV-PyMT tumors (Fig. 2I). Further, co-staining with CAF markers, showed DKK1 colocalization with elongated aSMA^+^ cells and partial colocalization with the more ubiquitous CAF marker COL1a1 (Fig. 2H, J). In concordance with its undetectable transcript expression, DKK1 staining was not present in the murine cancer lines.

Because in homeostatic conditions DKK1 is known to be highly expressed in bone by osteoblast-lineage cells^34^, we also evaluated expression of bone-derived *Dkk1* in mice bearing primary breast tumors. We found increased expression of *Dkk1* in crushed bones devoid of marrow cells of tumor bearing mice compared to no tumor controls (Supp Fig. 1C, F). These results indicate the existence of different sources of DKK1, with local production of DKK1 at tumor site by either tumor cells and/or CAFs, and distal production of DKK1 by bone cells.

### Bone and CAF-derived DKK1 contribute to systemic and local increases in DKK1 levels during tumor progression.

**Figure 3:**
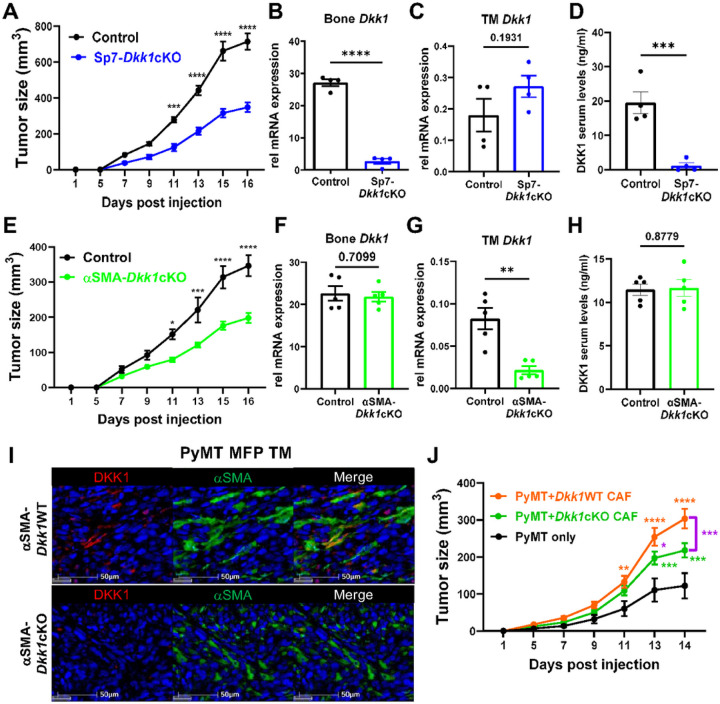
Bone and CAF-derived DKK1 contribute to systemic and local increases in DKK1 levels during tumor progression. (A) Tumor growth was determined by caliper measurements in 6–8 weeks old control and Sp7-Dkk1cKO mice (n= 4 mice/group) inoculated with PyMT in the MFP. (B, C) qRT-PCR for Dkk1 expression in bone and primary tumor. (D) DKK1 serum levels measured by ELISA. (E) Tumor growth was determined by caliper measurements in 10–12 weeks old control and aSMA-DkklcKO (n= 5 mice/group). (F, G) qRT-PCR for Dkk1 expression in bone and primary tumors. (H) DKK1 serum levels measured by ELISA. (I) Multiplex IHC of orthotopic PyMT tumors in aSMA-Dkk1 WT (top) or aSMA-Dkk1cKO mice (bottom) stained for DKK1 (red), aSMA (green), and hematoxylin (blue). (J) Tumor growth was determined by caliper measurements in WT mice co-injected with 105 PyMT cells and 105 tdT+ CAFs isolated from aSMA-Dkk1WT-tdT or aSMA-Dkk1cKO-tdT (n=8/group). Mice injected with tumor cells alone (n=4) were used as control. Results represent mean +/− SEM. Unpaired t-test (B-D, F-H), Two-way ANOVA followed by Bonferroni multiplecomparison test was used to determine significance (A, E, J). * P < 0.05, ** P < 0.01, *** P < 0.001 **** P < 0.0001.

To determine the role of bone versus CAF-derived DKK1 during tumor progression, we generated mouse models with targeted deletion of DKK1 in osteoblasts and fibroblasts. To specifically delete *Dkk1* from osteoblasts, we crossed *Dkk1*^*fl/fl*^ mice with the doxycycline-repressible Sp7 Cre line (herein referred to as Sp7-*Dkk1*cKO)^35^. Because *Dkk1* deletion leads to embryonic lethality, we fed moms and pups with a doxycycline-containing diet until weaning to suppress the transgene activation, and orthotopically injected the PyMT tumor cells when mice reached 6–8 weeks of age. Strikingly, Sp7-*Dkk1*cKO mice showed a significant reduction in primary tumor growth (Fig. 3A). While *Dkk1* expression at tumor site was not reduced compared to littermate controls, DKK1 levels in circulation were drastically reduced (Fig. 3B–D), indicating that bone-derived DKK1 contributes to systemic elevation of DKK1 during tumor progression.

Based on the expression of DKK1 in aSMA^+^ cells in the tumor microenvironment, next, we crossed *Dkk1*^fl/fl^ mice with the inducible aSMA CreER^T2^ line (referred to as aSMA-*Dkk1*cKO). We induced Cre activation by intraperitoneal administration of tamoxifen for 5 consecutive days (100mg/kg per dose) to 10–12 weeks old mice. As a control, we crossed the aSMACreER^T2^ mice with the Rosa26-LSL-tdTomato line (herein referred to as aSMA-tdT mice) and injected PyMT cells into their MFP to confirm the presence of aSMA-tdT^+^ cells exclusively at the tumor site, but not in the bone (Supp Fig. 3A). Intriguingly, aSMA-*Dkk1*cKO also showed to a significant reduction in primary tumor growth compared to littermate controls (Fig. 3E), despite showing efficient deletion of *Dkk1* only in the tumor mass but no changes in the bone and circulation (Fig. 3F–H). Multiplex IHC further confirmed expression of DKK1 or lack thereof in aSMA^+^ cells (Fig. 3i). To exclude the possibility of tamoxifen directly affecting tumor growth, we performed an MTT assay by culturing the PyMT tumor cells with different concentrations of tamoxifen (0, 1, 2, and 10 μM) for 24 and 48 hours. We did not observe any changes in cell density at all time points and doses tested (Supp Fig. 3B). To exclude the possibility that tamoxifen could affect PyMT tumor growth through suppression of estrogen, we injected the PyMT cells subcutaneously (SQ) into tamoxifen treated male mice and confirmed a significant reduction in tumor growth in aSMA-*Dkk1*cKO animals compared to controls (Supp Fig. 3C).

To further investigate the role of CAF-derived DKK1 during tumor progression, we co-injected *Dkk1*-deficient (*Dkk1*cKO) or sufficient (*Dkk*WT) CAFs with PyMT tumor cells (1:1 ratio) into the MFP of naive WT recipient mice. CAFs were isolated based on tdT expression from primary tumors in aSMA-Dkk1cKO-tdT and aSMA-*Dkk1*WT-tdT mice. Mice injected with tumor cells alone were used as controls. Highlighting the importance of local production of DKK1, mice co-injected with *Dkk1* deficient CAFs showed smaller tumor size compared to mice co-injected with *Dkk1* sufficient CAFs (Fig. 3J).

All together these results demonstrate that there are at least two sources of DKK1, one released by bone cells contributing to DKK1 levels in circulation and possibly exerting systemic pro-tumorigenic effects, and one from CAFs at tumor site, possibly affecting the local tumor microenvironment; abrogating DKK1 expression by either of the sources is sufficient to reduce tumor growth.

### DKK1 does not directly modulate the tumor cells.

To assess whether DKK1 exerts direct effects on tumor cell proliferation, we cultured the PyMT, 4T1, and EO771 tumor cells in the presence of recombinant DKK1 (rDKK1) at various concentrations (0, 50, 100, and 200 ng/ml) for 24, 48, and 72 hours and performed an MTT assay. For all three cell lines, rDKK1 did not increase cell density compared to unstimulated cells at all time points and doses tested (Supp Fig. 4A-C) nor induced any significant changes in cell cycle and survival (Supp Fig. 4D, E).

**Figure 4:**
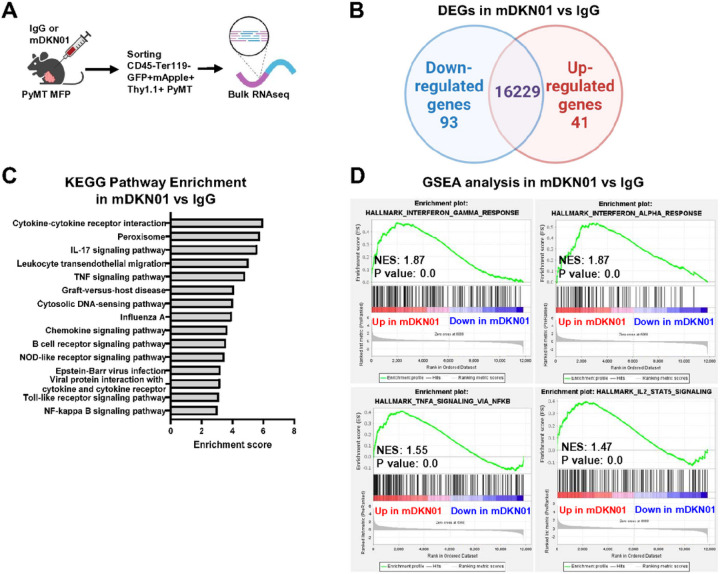
DKK1 does not directly support breast cancer cell growth. (A) Schematic representation of PyMT tumor cell isolation from IgG or mDKN01-treated mice and analysis of transcriptome via bulk RNA sequencing. (B) Venn diagram depicting uniquely and commonly expressed genes in PyMT cells isolated from orthotopic tumors injected into WT mice treated with IgG or mDKN01. (C) KEGG pathway enrichment analysis on differentially expressed genes (DEGs, p2). (D) GSEA analysis of hallmarks upregulated in PyMT tumor cells isolated from mDKN01-treated mice compared to IgG-treated mice.

To better understand how DKK1 promotes tumor progression *in vivo*, we performed bulk RNA sequencing of GFP-H2B-mApple-Thy1.1^+^ PyMT-BO1 cells isolated from orthotopic tumors in WT mice receiving IgG or mDKN01, after exclusion of Ter119^+^ erythrocytes and CD45^+^ immune cells (Fig. 4A, Supp Fig. 4F). Out of 16,363 genes sequenced, only 134 genes were differentially expressed (DEGs, p< 0.05 and |fold change|>2) between the two groups (Fig. 4B). KEGG pathway enrichment analysis confirmed no differences in pathways related to cell viability or cell cycle but rather showed changes in pathways related to immune responses (Fig. 4C). Gene set enrichment analysis (GSEA) further showed hallmarks of anti-tumor immune responses being upregulated in the mDKN01-treated tumors compared to IgG, including interferon-gamma response, interferon alpha response, TNFa signaling via NFkB, and IL-2/STAT5 signaling (Fig. 4D). These results suggest that expression of DKK1 at tumor site might contribute to an immune suppressive environment, rather than directly affecting tumor growth.

### Local production of DKK1 at tumor site affects tumor immune infiltration.

**Figure 5:**
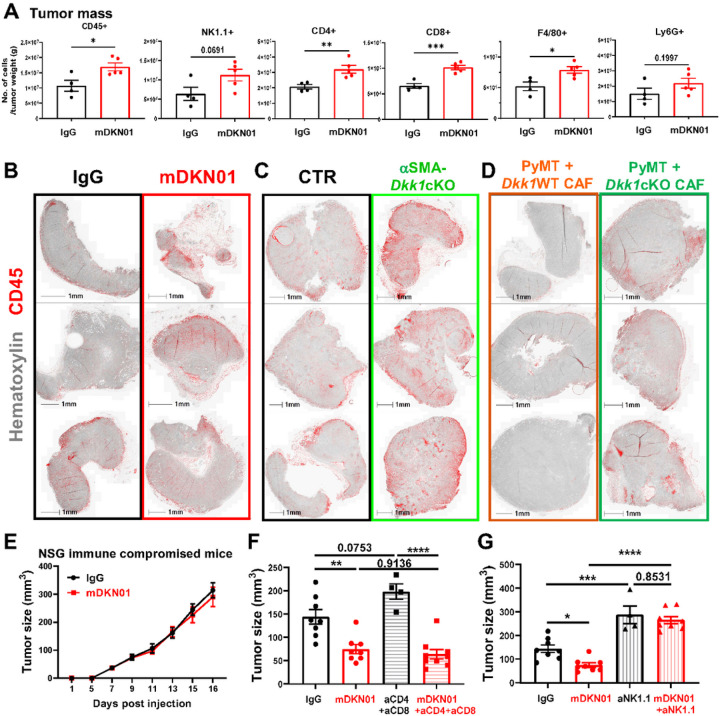
Local production of DKK1 at tumor site affects tumor immune infiltration. (A) Tumor infiltrating CD45+ immune cells, NK cells, T cells and myeloid subsets per grams of tumors from mice treated with IgG or mDKN01. (B-D) Deconvoluted IHC images from orthotopic PyMT tumors stained for CD45 (red) and hematoxylin (grey) isolated from WT mice treated with IgG or mDKN01 (B), aSMA-Dkk1WT and aSMA-Dkk1cKO mice (C), and mice co-injected with tumor cells and tdT+ CAFs from aSMA-Dkk1WT-tdT or aSMA-Dkk1cKO-tdT (D). (E) Tumor growth by caliper measurements in 6–8 weeks old NSG immune compromised mice (n= 6 mice/group) inoculated with PyMT into the MFP. (F, G) PyMT orthotopic growth determined by caliper measurements in 6–8 weeks WT mice treated with mDKN01 (10mg/kg) or IgG every other day along with anti-CD4 and anti-CD8 (F) or anti-NK1.1 (G) (n= 4–9 mice/group). Results represent mean +/− SEM. Unpaired t-test (A), Two-way ANOVA followed by Bonferroni multiple-comparison test was used to determine significance (E), Ordinary one-way ANOVA followed by Dunnett’s multiple-comparison test (F, G). * P < 0.05, ** P < 0.01, *** P < 0.001 **** P < 0.0001.

To determine if DKK1 modulates the immune landscape of the tumor microenvironment, we profiled the tumor-infiltrating immune populations from IgG or mDKN01-treated mice via flow cytometry, 16 days post tumor inoculation. We found a significant increase in the number of CD45^+^ cells per grams of tumors following mDKN01 administration, with CD4^+^ and CD8^+^ T cells, F4/80^+^ macrophages and NK cells being the most increased subsets (Fig. 5A, Supp Fig. 5A, B).

To determine whether local production of DKK1 at tumor site limits the infiltration of immune populations, we performed IHC to determine the localization of CD45^+^ cells in PyMT tumors isolated from IgG or mDKN01 treated mice (Fig. 5B), aSMA-*Dkk1*WT or aSMA-*Dkk1*cKO animals (Fig. 5C) and from WT mice co-injected with tumor cells together with aSMA-*Dkk1*WT or aSMA-*Dkk1*cKO CAFs (Fig. 5D). CD45^+^ cells primarily resided at the edges of the tumor mass in all control groups. In contrast, the presence of CD45^+^ immune populations in the central regions of the tumor mass was readily evident in animals following DKK1 neutralization or deletion of DKK1 in the CAFs. These results suggest that CAF-derived DKK1 can limit the infiltration of immune cells at tumor site, regardless of the elevated levels of DKK1 in circulation.

### DKK1 targets NK cells to support tumor progression.

To identify the immune populations targeted by DKK1, we injected PyMT cells into the MFP of female NSG mice, which lack T, B, and NK cells, and administered IgG or mDKN01. Strikingly, the anti-tumor effects of mDKN01 were fully abrogated in this mouse model (Fig. 5E). Next, we adopted a selective immune cell depletion approach. First, we depleted T cells by administering anti-CD4 and anti-CD8 antibodies, delivered every 4 days starting 2 days before tumor inoculation, using 500μjg for the first dose and 250μjg for the subsequent doses, in mice orthotopically injected with PyMT cells and concomitantly receiving IgG or mDKN01. As expected, depletion of T cells only slightly increased tumor burden compared to the IgG control group (Fig. 5F, Supp Fig. 5C), confirming the low involvement of T cells in the PyMT tumor model^36^. Furthermore, mDKN01 treatment significantly reduced tumor growth in mice depleted of T cells to levels comparable to mDKN01 as single agent, suggesting that T cells are not targeted by DKK1.

Next, we depleted NK cells by administering the anti-NK1.1 antibody (100μjg/dose) once a week starting 2 days before tumor inoculation, in mice orthotopically injected with PyMT cells receiving IgG or mDKN01. Unlike the T cells, depletion of NK cells significantly increased tumor burden compared to control mice and completely abrogated the anti-tumor effects of mDKN01 (Fig. 5G, Supp Fig. 5D). These results indicate that DKK1 promotes tumor growth by impacting NK cells, but not T cells, in the PyMT breast cancer model.

### DKK1 suppresses perforin-mediated NK ceil cytotoxicity.

**Figure 6:**
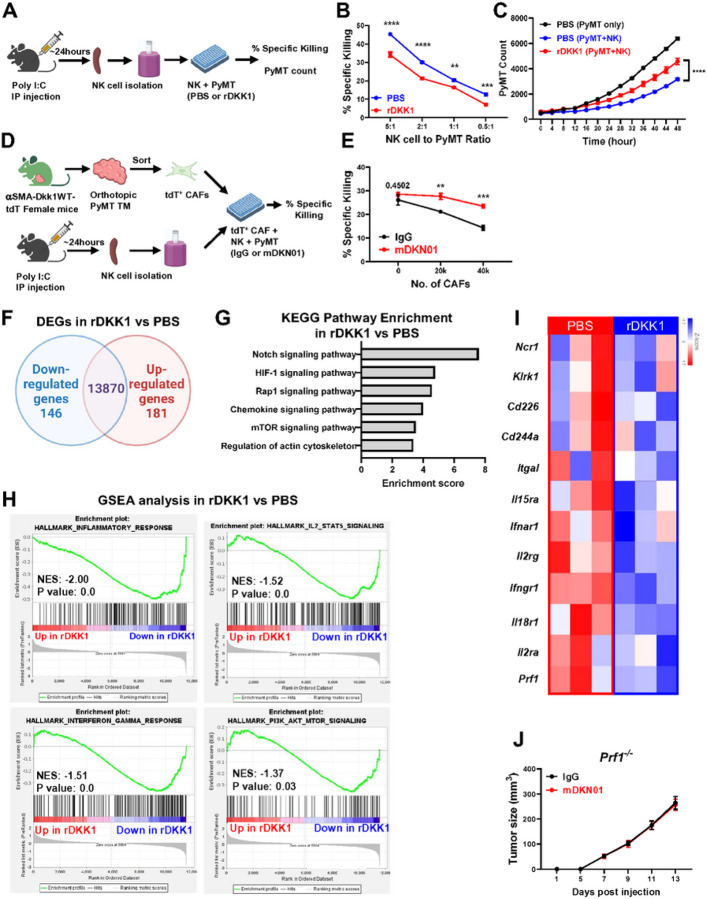
DKK1 suppresses NK cell cytotoxicity. (A) Schematic representation of NK cell isolation from spleen of Poly I:C treated mice and incubation with PyMT target cells in the presence of PBS or rDKK1. (B, C) Analysis of % of killed PyMT target cells after 4 hours incubation with NK cells (B) or remaining mApple+ PyMT-BO1 target cells over indicated time (C). (D, E) Schematic representation of CAF isolation from orthotopic PyMT tumors in aSMA-DKK1WT-tdT mice and isolation of NK cells. NK cells and increasing number of CAFs were incubated with PyMT target cells in the presence mDKN01 or IgG (D) and % of killed target cells measured 4 hours later (E). (F) Venn diagram indicating numbers of uniquely and commonly expressed genes in NK cells isolated spleen of Poly I:C treated mice (n=3) and stimulated with rDKK1 (200ng/ml) or PBS as a control (n=3/group). (G) KEGG pathway enrichment analysis showing differentially expressed genes (DEGs, p<0.05, fold change|>2). (H) GSEA analysis showing hallmarks downregulated in rDKK1 stimulated NK cells. (I) Heatmap of genes related to NK cell cytotoxicity in PBS or rDKK1 stimulated NK cells. (J) Tumor growth was determined by caliper measurements in WT and Prf1−/− mice (n=4 mice/group) inoculated with PyMT into the MFP and treated i.p. with mDKN01 (10mg/kg) or control IgG antibody every other day. Results represent mean +/− SEM. Experiments in (B, C and E) were performed in triplicates. Two-way ANOVA followed by Bonferroni multiple-comparison test was used to determine significance (B, C, E, J). ** P < 0.01, *** P < 0.001 **** P < 0.0001.

To determine whether DKK1 directly affects NK cell functionality, we performed *ex vivo* killing assays to quantify NK cell-mediated killing of PyMT tumor cells in the presence and absence of rDKK1. NK cells were isolated from spleens of poly I:C treated mice and co-cultured with the cell trace violet (CTV)-labeled PyMT tumor cells for 4 hours. Tumor cell death was assessed by the expression of 7-AAD via flow cytometry (Fig. 6A, Supp Fig. 6). rDKK1 significantly suppressed NK cell mediated PyMT killing at all tested effector (NK cells) to target (PyMT) ratios (Fig. 6B). To assess integrated killing overtime, we performed a 48-hour IncuCyte Live Cell assay using a 2:1 effector (NK cells) to target (H2B-mApple-Thy1,1^+^ PyMT-BO1 cells) ratio (Fig. 6A). Addition of rDKK1 resulted in a significantly higher number of tumor cells at the end of assay, indicating impaired NK cell killing of PyMT tumor cells (Fig. 6C).

To further investigate whether CAFs at the tumor site suppress NK cell functions by producing DKK1, we co-cultured tdT^+^ CAFs isolated from orthotopic tumors in aSMA-*Dkk1*WT-tdT mice, together with NK cells and CTV-labeled PyMT tumor cells in the presence of IgG or mDKN01 (Fig. 6D). Increasing the number of CAFs reduced the NK-mediated killing of PyMT tumor cells. While DKK1 neutralization had no effects in the absence of CAFs, it restored NK cell functionality against PyMT cells in the presence of CAFs (Fig. 6E). These results demonstrate that CAF-derived DKK1 suppresses NK cell tumoricidal activities in the tumor microenvironment.

To determine how DKK1 impacts NK cells at the transcriptional level, we performed bulk RNA sequencing of NK cells sorted from the spleen of poly I:C treated mice using the CD45^+^CD3^−^NK1.1^+^ markers and stimulated *ex vivo* with rDKK1 for 4 hours. Out of 14,197 genes detected, 327 genes were differentially expressed in the rDKK1-stimulated versus unstimulated NK cells (Fig. 6F). KEGG pathway analysis showed enrichment in signaling pathways related to NK cell development (Notch signaling pathway) and function (HIF-1, Rap1, mTOR and pathways involved in the regulation of actin cytoskeleton) (Fig. 6G). Furthermore, GSEA analysis showed reductions in genes related to anti-tumor immune responses including the IL-2/STAT5 pathway, the interferon-gamma response and the PI3K/AKT/mTOR signaling (Fig. 6H). Accordingly, the rDKK1-exposed NK cells showed decreased gene expression of cytokine receptors *Ifngrl, tfnarl, il2r, Il15ra* and il18r1, adhesion molecules involved in the maintenance of the immunological synapse *Itgal, Cd244a*, and *Cd226* and activating receptors *Ncr1, Klrkl* and *Cd226* (Fig. 6I). We also observed that the expression of *Prf1*, a key effector molecule used by NK cells to make pores and transfer cytotoxic granules to the target cells to induce their specific killing, was also decreased in the NK cells exposed to rDKK1.

To further investigate whether DKK1 impairs NK cell-mediated cytotoxicity via perforin downregulation, we injected the PyMT cells into the MFP of mice lacking perforin (*Prf1*^−/−^) treated with IgG or mDKN01. Strikingly, the anti-tumor effects of mDKN01 were fully abrogated (Fig. 6J), similarly to what observed in the NSG model (Fig. 5E). Since T cell depletion did not alter the anti-tumor effects of mDKN01 (Fig. 5F), this suggests that loss of perforin primarily impacts NK cell driven responses in this context. Thus, these data demonstrate that local production of DKK1 at tumor site promotes tumor growth by suppressing perforin-mediated NK cell cytotoxicity.

### DKK1 directly suppresses human NK cell functions.

**Figure 7:**
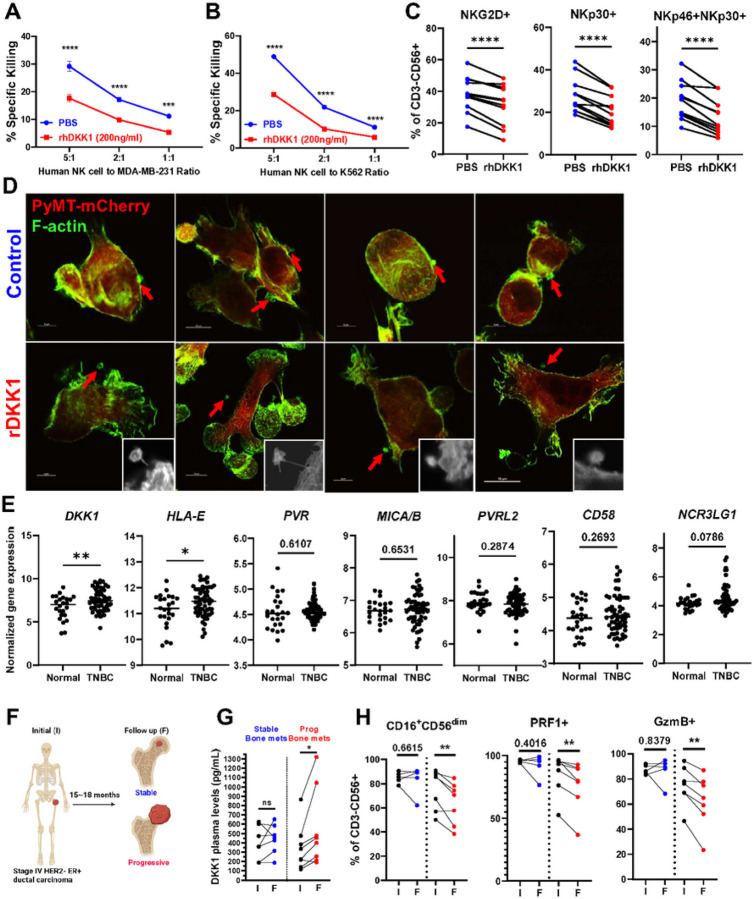
DKK1 suppresses human NK cells and its levels are increased with progression of bone metastases. (A, B) Analysis of % of killed MDA-MB231 human breast cancer cells (A) and K562 (B) target tumor cells in the presence of rhDKK1 (200ng/ml) or PBS as a control. (C) Flow cytometric analysis of NK cell activating ligands on human NK cells from different donors stimulated with rhDKK1 for 24 hours or PBS.(D) Representative immunofluorescence images of mCherry+ PyMT cells (Red) cultured with murine NK cells in the presence of PBS or rDKK1 for 3 hours prior to fixation and stained for F-actin (Green). Small, mCherry-cells visualized by red arrows or by image contrast in insets represent NK cells. Images in insets are enlarged 1.5 times. (E) Normalized gene expression of DKK1 and NK cell ligands in human triple-negative breast cancer versus normal breast tissues (GSE21653). (F) Schematic representation of blood sample collection of advanced breast cancer patients at time of diagnosis of bone metastases and at 15–18 months follow-up visit receiving standard-of-care and antiresorptive (Denosumab) treatments. (G, H) DKK1 plasma levels, and % of NK cell subsets in patients with regressive/stable (blue) versus progressive bone metastases (red) from initial diagnosis (abbreviated as I) and follow-up visits (abbreviated as F). Results represent mean +/− SEM (A, B). Two-way ANOVA followed by Bonferroni multiple-comparison test was used to determine significance (A, B), unpaired t-test (E), and paired t-test were used to determine significance (C, G, H). * P < 0.05, ** P < 0.01, *** P < 0.001 **** P < 0.0001.

To investigate whether DKK1 exerts similar suppressive effects on human NK cells (hNK cells), we collected peripheral blood from healthy donors and isolated hNK cells to assess their ability to kill the MDA-MB-231 human breast cancer line in the absence or presence of recombinant human DKK1 (rhDKK1). Consistent with the murine NK cell findings (Fig. 6B, C), rhDKK1 significantly decreased hNK cell killing of MDA-MB-231 cells (Fig. 7A) and of the NK-sensitive K562 target cell line (Fig. 7B).

We next hypothesized that one mechanism through which DKK1 suppresses NK cell function is by modulating NK cell activating receptor expression. Indeed, rhDKK1 led to a significant decrease in NK activating receptors such as NKG2D, NKp30, and NKp46 (Fig. 7C, Supp Fig. 7A). Based on these findings, we considered whether DKK1 may be impacting the ability of NK cells to interact with the tumor cells. Murine NK cells, isolated from the spleen of poly I:C treated mice, and mCherry^+^ PyMT cells were plated at 2:1 ratio in the presence or absence of rDKK1 (200ng/ml) for 3 hours, followed by F-actin staining and analyzed by high magnification confocal microscopy. We only evaluated interactions between tumor and NK cells in proximity to each other and excluded cells that were more than 10μm apart. In control conditions, 70% of NK cells were found in direct association with the tumor cells (8/11) (Fig. 7D top). In the presence of rDKK1, only 33% of NK cells were in close contact with the tumor cells (3/9), while the majority of NK cells were in adjacent regions interacting with the tumor cells through a long protrusion (Fig. 7D bottom).

Finally, to explore the possibility that DKK1 might modulate the expression of NK cell activating and/or inhibitory ligands on the tumor cells, we stimulated the MDA-MD-231 breast cancer line with rhDKK1 but noted no changes in activating and inhibitory NK ligand levels via flow cytometry (Supp Fig. 7B, C) and qRT-PCR (Supp Fig. 7D). These results were also confirmed in a microarray dataset from human triple-negative breast cancer samples (GSE21653^37^). Although gene expression levels of *DKK1* were significantly higher in TNBC samples compared to normal breast tissue, only *HLA-E*, whose binding to NKG2A has been shown to disrupt actin formation at the immunological synapse and negatively affect NK function^38^, showed increased expression in TNBC (Fig. 7E). Expression of the NK activating ligands *PVR, MICA/B, PVRL2, CD58*, and *NCR3LG1* were not statistically different. Collectively, these findings suggest that in human breast cancer, DKK1 directly suppresses NK cells, rather than exhibiting direct effects on tumor cells.

### DKK1 levels correlate with metastatic progression and reduced cytotoxic NK cells in breast cancer patients.

Finally, to determine whether DKK1 levels correlate with tumor progression and immune suppression in breast cancer patients, we analyzed DKK1 plasma levels and the activation status of NK cells in the blood of 15 patients with stage IV, HER^−^, ER^+^ breast cancer and skeletal disease, at time of diagnosis and after 15 to 18 months of standard-of-care endocrine therapy-based regimens with denosumab as antiresorptive therapy. Skeletal metastases were monitored by routine surveillance imaging with CT and bone scintigraphy scans. 7 patients were classified as stable as they had no radiographic evidence of skeletal metastatic progression during the study period. 8 patients were classified as progressive as they demonstrated radiographic evidence of new or progressive skeletal lesions during the study period (Fig. 7F, Table. 1). Although DKK1 levels in circulation were not significantly different in the stable and progressive patients at baseline, patients with progressive skeletal metastases demonstrated a significant increase in DKK1 compared to time of diagnosis (Fig. 7G).

To assess whether patients with increased DKK1 had reduced and/or dysfunctional NK cells, we measured the number of CD3^−^CD56^+^ NK cells in circulation and found no changes between the stable and progressive patients (Supp Fig. 7E, F). However, the percentage of circulating CD16^+^CD56^dim^ NK cells, which represent the more cytotoxic subset^39^, were reduced in patients with progressive disease (Fig. 7H) as the disease progressed. These cells also showed lower expression of perforin and granzyme B as the disease progressed. These results suggest that patients with progressive bone metastatic disease experience systemic immune suppression accompanied by increased DKK1 in circulation and decreased cytotoxic NK cells.

## Discussion

In this study, we demonstrate that high levels of DKK1 in breast cancer creates an immune suppressive microenvironment through direct inhibition of NK cell tumoricidal functions. Targeting DKK1 as single therapy with a monoclonal antibody has significant anti-tumor effects. Moreover, this study demonstrates the previously unappreciated impact of DKK1 on systemic and local immune suppression in breast cancer. Bone cells contribute to the elevated levels of circulating DKK1 in response to a distant tumor, while CAFs produce DKK1 at tumor site and do not affect DKK1 levels systemically. Intriguingly, deletion of either source of DKK1 results in a significant anti-tumor effect. Our findings indicate the importance of monitoring both systemic and tumor DKK1 levels in breast cancer patients and warrants the exploration of neutralizing DKK1 to achieve an efficient therapeutic response. These results position DKK1 and its inhibitory effects on NK cells as a potentially important driver of breast cancer progression.

Breast cancer is poorly immunogenic, and hence responds poorly to immune-based anti-tumor therapies. We now find that local production of DKK1 at tumor site limits tumor immune infiltration, while DKK1 neutralization or its deletion in CAFs reverses these effects. Differently from melanoma, where anti-DKK1 antibody requires functional T cells^23^, in breast cancer T cell depletion does not majorly affect tumor growth or impact the therapeutic efficacy of mDKN01. Results from our mouse models are in line with the numerous inconclusive clinical trials in ER^+^ breast cancer patients showing equivocal activity of T cell-based therapies to improve the pCR or disease free survival^5^. In contrast, we identify NK cells as important modulators of the anti-tumor effects of mDKN01 in our murine breast cancer model. Our findings are in line with the observation that the intratumoral abundance of NK cells correlate with increased pCR rate in breast cancer patients undergoing neoadjuvant chemotherapy^10^.

Direct effects of DKK1 on NK cell function have never been reported. In this study, we demonstrate that DKK1 suppresses NK cell functionality. Short term incubation of murine NK cells with rDKK1 induces changes in their transcriptional profile associated with decreased IL-2/STAT5, IFN-gamma and PI3K/AKT pathways, all known to be required for NK cell activation. The lower levels of perforin, adhesion molecules and pathways related to cytoskeletal reorganization from our RNA sequencing and flow cytometry analyses further suggest defects in the formation of immunological synapses with their target cells. Indeed, immune fluorescence images show reduced interactions between NK and tumor cells in the presence of DKK1. These findings are further supported by the observation that mDKN01 loses its therapeutic efficacy in perforin-deficient mice which have no other known dysfunctions other than granule-mediated cytotoxicity^40^. Consequently, the killing efficiency of murine and human NK cells is significantly reduced by DKK1. Similarly, DKK1 producing CAFs reduce the NK cell cytotoxicity, which can be restored by DKK1 neutralization. Therefore, DKK1 levels should be monitored in future clinical trials consisting of NK cell activating therapies due to its potential NK cell suppressive effects that were not previously appreciated.

Interestingly, we do not observe modulation of NK cell activating ligands on the tumor cells, neither *in vivo* nor *in vitro*. This finding differs from the effects reported on disseminated stem-like breast cancer cells in the lung where DKK1 induced evasion from NK cell immunity via downregulation ULBP ligands^41^, and to support metastatic outgrowth through increased expression of SLC7A11 and protection from lipid peroxidation and ferroptosis^42^. We also did not find any direct effects of DKK1 on breast cancer cell proliferation and/or survival. It is possible that direct pro-tumorigenic effects of DKK1 on breast cancer only occur at the level of the stem cells, which are too few in our model. Thus, targeting DKK1 in breast cancer could offer multiple therapeutic benefits from increasing anti-tumor immunity via unleashing NK cell suppression from the tumor microenvironment to targeting disseminated tumor cells and/or cancer stem cells.

Bone is a preferred organ for metastatic dissemination in breast cancer. We find DKK1 to be highly expressed in osteolineage cells and that deletion of bone-derived DKK1 exerts profound anti-tumor effects. At the moment it is not clear if and how bone-derived DKK1 affects NK cells. Since bone is the major site for hematopoiesis, we cannot exclude that bone-derived DKK1 might reduce the number of NK cell progenitors and reprogram hematopoiesis towards myelopoiesis. Findings from mice overexpressing DKK1 in bone cells confirmed defective hematopoietic stem cell transplant^43^, thus suggesting that DKK1 could lead to hematopoietic reprogramming towards immune suppression during tumor progression. We also find DKK1 to be increased in breast cancer patients with progressive bone metastatic disease compared to those with stable disease. Importantly, the percentages of cytotoxic NK cells are reduced in the progressive patients, confirming our finding in the mouse model. These results are also concordant with a previous study showing increased metastatic dissemination to bone in DKK1-expressing tumors^29^. However, the same study showed that DKK1 increased metastatic dissemination to bone via activation of osteoclast-mediated bone resorption. In contrast, our clinical findings show that high levels of DKK1 correlate with progression of bone metastatic disease despite osteoclast blockade, since patients were receiving anti-resorptive treatment, suggesting that DKK1-mediated immune suppression is a key driver of metastases to bone.

In sum, we find that DKK1 is an important suppressor of NK cell activation and function in breast cancer and that its targeting reduces tumor progression in multiple mouse models. Considering the promising therapeutic effects of DKK1 neutralization in patients with gastric and endometrial cancer (NCT04363801, NCT03395080), DKK1 targeting should be investigated as a therapeutic option for patients with breast cancer.

## Methods

### Cell lines

Polyoma middle tumor-antigen murine mammary tumor cells (PyMT, C57BL/6), mCherry-conjugated PyMT (PyMT-mCherry; generously provided by David DeNardo, Washington University in St. Louis, MO), PyMT-derivative PyMT-BO1 conjugated with firefly luciferase (PyMT-BO1-fluc; generously provided by Katherine Weilbaecher, Washington University in St. Louis, MO), and H2B-mApple and Thy1.1 conjugated PyMT-BO1 (PyMT-BO1-GFP-fluc-H2B-mApple-Thy1.1; generously provided by Sheila Stewart, Washington University in St. Louis, MO), EO771 murine mammary tumor cells (EO771-fluc, C57BL/6; generously provided by Sheila Stewart, Washington University in St. Louis, MO), 4T1 murine mammary tumor cells (4T1-fluc, BALB/c; generously provided by David Piwnica-Worms, The University of Texas MD Anderson, Houston TX), and MDA-MB-231 human breast cancer cells (ATCC) were cultured at 37°C with 5% C0_2_ in complete media (DMEM supplemented with 100 μg/ml streptomycin, 100 IU/ml penicillin, and 1 mM sodium pyruvate) containing 10% FBS. K562 cell line (ATCC) was cultured in complete media (RPMI 1640 supplemented with 2 mM L-glutamine, 100 μg/ml streptomycin, 100 IU/ml penicillin, 1X nonessential amino acids, and 1 mM sodium pyruvate) containing 10% FBS. All cell lines were tested for *Mycoplasma* every 2 months. Aliquots for each cell line were used for maximum 1 month after initial thaw.

### Animal models

Female wild-type (WT) C57BL/6 (The Jackson Laboratory (JAX), #000664), WT BALB/c (JAX #000651), B6(Cg)-7/A^c_2J^/J (albino C57BL/6, JAX #000058), NOD.Cg*-Prkdc*^*scid*^*Il2rg*^*tm1Wji*^*/SzJ* (NSG, JAX #005557), *C57BL/6-Prf1*^*tm1sdz*^*/J (Prf1*^*−/−*^, JAX #002407), Sp7 Cre (Sp7-tTA, tetO-EGFP/Cre, JAX #006361) mice were purchased from The Jackson Laboratory. Mice arrived at 4–6 weeks of age and were allowed to recover from shipping stress and acclimatize to the new environment for at least 2 weeks before use in experiments. aSMACreER^T2^ transgenic mice were a generous gift from Dr. Ivo Kalajzic (University of Connecticut Health Center, Farmington, CT^44^). *Dkk1* floxed mice were a generous gift from Seppo J. Vainio (University of Oulu, Finland^45^). In experiments using conditional KO mice, *Cre*^+^*,Dkk1*WT or Cre^−^;*Dkk1*^fl/fl^ were used interchangeably as littermate controls. Animals were housed in a pathogen-free animal facility at Washington University (St. Louis, MO). Female littermate mice were used in all experiments according to protocols approved by the Institutional Animal Care and Use Committee.

To establish tumors, PyMT, PyMT-BO1-fluc, PyMT-BO1-GFP-fluc-H2B-mApple-Thy1.1, EO771-fluc, and 4T1-fluc tumor cells were suspended in 1:1 PBS/Matrigel ratio (Corning 354234) and injected into the mammary fat pad (10^5^ cells, MFP) or inoculated subcutaneously (10^5^ cells, SQ) in the left flank, or resuspended in PBS and injected into the right tibia (10^4^ cells, i.t.) or intracardiacally (10^4^ cells, i.e.) of sex- and age-matched mice. For aSMACreER^T2^ transgenic mice, tamoxifen (Sigma, 100mg/kg) resuspended in corn oil was intraperitoneally injected for 5 consecutive days. For MFP and SQ tumors, measurements were performed every other day with a caliper and volumes were calculated using the following formula: V = 0.5 (length [mm] × width [mm]^2^). For i.t. and i.c. tumors, growth curves were determined by bioluminescence imaging.

### Bioluminescence imaging (BLI)

BLI was performed as previously described^46^. In brief, tumor growth was monitored by BLI using an IVIS 50 imaging system (PerkinElmer, 1–60 second exposures, binning 4, 8, or 16, FOV 15cm, f/stop1, open filter). Mice were i.p. injected with D-luciferin (150mg/kg in PBS; Gold Biotechnology) and imaged 10 minutes later under isoflurane anesthesia (2% vaporized in O_2_). Bioluminescence photon flux (photons per second) data were analyzed by region of interest measurements (fixed region of interest over the whole body, or hindlimb) in Living Image 3.2 (Caliper Life Sciences). For i.c. injections, mice with extra pleural intrathoracic tumors were excluded from analysis.

### ELISA

Serum or plasma levels of DKK1 from mice or patients were quantified using ELISA kits specific for DKK1 (Mouse Dkk-1 Quantikine ELISA kit or Human Dkk-1 Quantikine ELISA kit, R&D systems) as per the manufacturer’s protocols.

### In vivo treatments (Neutralizing antibodies)

The *in vivo* monoclonal Ab anti-mouse anti-DKK1 Ab (Leap therapeutics, mDKN-01) was used to neutralize DKK1 and mouse lgG2a isotype control (FcR incompetent construct (D265A), clone 4-4-20, Absolute antibody) was used as antibody control. Treatment consisted of intraperitoneal (i.p.) injections of anti-DKK1 and control antibodies at a concentration of 10 mg/kg three times a week.

The monoclonal anti-mouse anti-CD4 Ab (BioXCell, clone GK1.5), anti-mouse anti-CD8a (BioXCell, clone 2.43) were used to deplete T cells and PBS was used as control. Treatment consisted of intraperitoneal (i.p.) injections of anti-CD4 and anti-CD8a into mice 2 days prior to tumor cell implantation using the following regimen: 500μg on the first dose and 250μg for the subsequent doses every 4 days of each antibody.

The monoclonal Ab anti-mouse anti-NK1.1 Ab (Leinco Technologies, Inc., clone PK136), was used to deplete NK cells and PBS was used as control. Treatment consisted of intraperitoneal (i.p.) injections of anti-NK1.1 into mice 2 days prior to tumor cell implantation 100μg once a week. Effective depletion of T and NK cells were routinely assessed by flow cytometry of peripheral blood.

### Human study

All human samples were obtained in accordance with guidelines set by the Institutional Review Board of Washington University (IRB ID#: 201102244) and followed federal and state guidelines. All participants gave written informed consent under the IRB-approved protocol prior to inclusion in the study, including access of archival tumor tissue for research. Samples were deidentified prior to sharing with collaborators. All studies were conducted in compliance with the Declaration of Helsinki. Patients had diagnosis of estrogen receptor (ER)-positive, HER2-negative breast cancer, stage IV, with bone metastases, prior to first line systemic therapy for metastatic breast cancer or had prior therapy for metastatic breast cancer but met the following criteria: (i) no prior chemotherapy or immune therapy in the past 2 months, (ii) patients currently stable or progressing on hormonal therapy or hormonal therapy combination or starting hormonal therapy or hormonal therapy combination, and (iii) no limitation on the number of prior hormonal therapy or chemotherapy treatments. Radiologic tumor assessment was required within 1 month prior to or after the collection of the baseline blood sample to serve as the baseline tumor assessment. Exclusion criteria included: uncorrected coagulopathy, bleeding tendency, or other conditions that might increase the risk of a biopsy, blood draw, or other procedure; any reason that would make the patient unlikely to comply with study requirements or be incapable of providing appropriate consent (e.g., confusion, infirmity, alcoholism, etc.). Prior history of other invasive malignancies was not an exclusion criterion, unless the disease was active and progressing at the time of protocol screening.

### Human sample collection and processing

Peripheral blood was collected at enrollment and at 15–18 months follow-up visit from 15 patients with stage IV HER2-negative, ER-positive ductal carcinoma with established skeletal metastases being treated with denosumab (anti-RANKL) and on standard-of-care treatment under an IRB approved protocol (IRB ID#: 201102244). To isolate peripheral blood mononuclear cells (PBMC), EDTA-treated whole blood was diluted to a volume of 20 mL with PBS, transferred to a 50 mL conical tube, and underlaid with 15 mL of Ficoll (Atlantal Biologicals). Tubes were centrifuged at 400 × g for 30 minutes. The PBMC fraction was collected at the interface layer and washed three times with 40mLof PBS. After counting, a minimum of 5×10^6^ PBMCs were frozen in 10% [volume for volume (v/v)] DMSO (Sigma-Aldrich, catalog no. D5879) in FBS and stored in liquid nitrogen for subsequent analysis.

### Human NK cell purification and cell culture

Human platelet apheresis donor PBMCs were obtained by Ficoll centrifugation. NK cells were purified using RosetteSep (StemCell Technologies, ≥ 95% CD3^−^CD56^+^). Cells were plated at 3–5×10^6^ cells/mL and cultured in complete RPMI 1640 medium containing 10% human AB serum (Sigma-Aldrich) supplemented with rhlL-15 (1 ng/mL) to support survival, with 50% of the medium being replaced every other day with fresh cytokines.

### scRNAseq analysis

For scRNA-seq analysis of the human breast cancer dataset, the matrix was downloaded from the European Genome-Phenome Archive (EGA) EGAS00001005173 and analyzed using Seurat version 4. For the Seurat object, genes expressed by less than 3 cells and cells expressing less than 200 or more than 6000 genes were excluded. Log-based normalization (NormalizeData function) was performed to normalize the expression matrix, FindVariableFeatures, and ScaleData arguments were then applied to the dataset. Principle component analysis (PCA) and adaptively-threshold low-rank approximation (ALRA) were conducted as well^47^. UMAP dimensional reduction was performed using the first 20 PCA components. We then conducted FindNeighbors and FindClusters functions to cluster cells at a resolution of 0.1. The FindAllMarkers function was used to identify signature genes for each cluster. Non-tumor (negative for KRT8, 18, 14, 17, and EPCAM) non-immune cell populations were re-clustered for in-depth analysis of endothelial cells (positive for PECAM1) and CAF subpopulations (positive for PDGFRB). All visualizations of gene expression were performed based on the ALRA assay of the dataset.

### Bulk RNAseq analysis

RNA was isolated from the sorted cells using RNeasy Micro Kit (Qiagen). Total RNA integrity was determined using Agilent Bioanalyzer or 4200 Tapestation. Library preparation was performed with 10ng of total RNA with a Bioanalyzer RIN score greater than 8.0. ds-cDNA was prepared using the SMARTer Ultra Low RNA kit for Illumina Sequencing (Takara-Clontech) per manufacturer’s protocol. cDNA was fragmented using a Covaris E220 sonicator using peak incident power 18, duty factor 20%, cycles per burst 50 for 120 seconds. cDNA was blunt ended, had an A base added to the 3’ ends, and then had Illumina sequencing adapters ligated to the ends. Ligated fragments were then amplified for 12–15 cycles using primers incorporating unique dual index tags. Fragments were sequenced on an Illumina NovaSeq-6000 using paired end reads extending 150 bases. Partek Flow software (Partek Inc., St. Louis, MO) was used for data analysis. Briefly, sequenced reads were aligned with STAR 2.7.8a index (Mus musculus - mm10 assembly, Whole genome index). Raw read counts were obtained by quantitating aligned reads using HTSeq with Ensembl Transcripts release 102 annotation model. Raw read counts were then normalized using counts per million (CPM) and offset of 0.0001 was added to all normalized read counts. Normalized read counts for each mRNA were statistically modeled using Partek Flow’s Gene Specific Analysis (GSA) approach. Differentially expressed genes (DEGs) were then filtered by using P-values less than or equal to 0.05 and fold changes bigger than 2. GSEA analysis was performed as previously described^48, 49^. The data discussed in this publication have been deposited in NCBI’s Gene Expression Omnibus^50^ and are accessible through GEO Series accession number GSE 262733 (https://www.ncbi.nlm.nih.gov/geo/query/acc.cgi?&acc=GSE262733).

### Real-time PCR analysis

RNA was isolated from the cell line or tumor mass using RNeasy Mini Kit (Qiagen). Purified RNA was then reverse transcribed to cDNA using High Capacity cDNA reverse transcription kit (Applied Biosystems) according to the manufacturer’s instructions. The subsequent real-time PCR analysis was performed with SYBR Green PCR Master Mix (Applied Biosystems) and primers specific for murine DKK1 and cyclophilin were used as follow: for *Dkk1*, CTC ATC AAT TCC AAC GCG ATC A (forward), GCC CTC ATA GAG AAC TCC CG (reverse) and for *cyclophilin* AGC ATA CAG GTC CTG GCA TC (forward) and TTC ACC TTC CCA AAG ACC AC (reverse).

For primers specific for human NK cell activating/inhibitory receptor ligands were used as follow: for *HLA-E*, TTC CGA GTG AAT CTG CGG AC (forward), GTC GTA GGC GAA CTG TTC ATA C (reverse), *PVR*, GGA CGG CAA GAA TGT GAC CT (forward) GGT CGT GCT CCA ATT ATA GCC T (reverse), *MIC-A*, CTT CAG AGT CAT TGG CAG ACA T (forward), TGT GGT CAC TCG TCC CAA CT (reverse), *PVRL2*, CAC TTG CGA GTT TGC CAC C (forward), GCC ACT GTC GTA GGG TCC T (reverse), *CD58, AGA GCA* TTA CAA CAG CCA TCG (forward), ATC TGT GTC TTG AAT GAC CGC (reverse).

### Histology

Freshly isolated mouse primary tumors were fixed in 10% neutral-buffered formalin (DiRuscio & Associates, Inc.) for 24 hours. Tissues were paraffin-embedded and sectioned 5μm thick by the histology core of the Washington University Musculoskeletal Research Center. Tissues were automatically stained via the Bond Rxm (Leica Biosystems) following dewaxing and appropriate epitope retrieval. Immunostaining was chromogenically visualized using the Bond Polymer Refine Detection (#DS9800, Leica Biosystems) or the Bond Polymer Refine Red Detection (#DS9390, Leica Biosystems). Slides were mounted using Xylene-based Cytoseal (Thermo Fisher) or Vectamount (Vector Labs) as appropriate. Next, slide images were scanned via a Zeiss AxioScan 7 microscope. All IHC analyses were performed on the HALO image analysis platform (Indica Labs, Deconvolution v1.1.1, Multiplex IHC v.3.2.3 algorithms).

Human tissue microarrays were prepared by The St. Louis Breast Tissue Registry (funded by The Department of Surgery at Washington University School of Medicine, St. Louis, MO). Data and tissues were obtained in accordance with the guidelines established by the Washington University Institutional Review Board (IRB #201102394) and WAIVER of elements of Consent per 45 CFR 46.116 (d). All patient information was de-identified prior to sharing with investigators. All human research activities and all activities of the IRBs designated in the Washington University (WU) Federal Wide Assurance (FWA), regardless of sponsorship, are guided by the ethical principles in “The Belmont Report: Ethical Principles and Guidelines for the Protection of Human Subjects Research of the National Commission for the Protection of Human Subjects of Biomedical and Behavioral Research”.

**Table:** 

Reagent	Source	Dilution
aSMA	Abcam (ab5694)	1:1500 (Human), 1:200 (Mouse)
COL1a1	Cell signaling (72026)	1:100 (Mouse)
DKK1	Proteintech (21112–1-AP)	1:100 (Human), 1:3000 (Mouse)
PDGFRa	Cell signaling (5241)	1:200 (Human)
PanCK	Novus (NBP2–29429)	1:1000 (Human)

### In vitro assays

5×10^2^ PyMT or 4T1 cells and 2×10^3^ EO771 cells were plated for 24 hours before treatment with rDKK1 (50, 100, 200 ng/ml) or tamoxifen (1, 2, 10 μM) for indicated amount of time. The 3-(4,5-dimethylthiazol-2-yl)-2,5-diphenyltetrazolium bromide (MTT) assay (Invitrogen) and annexin V assay (ThermoFisher Scientific) were performed per manufacturer’s protocol.

### Multiparametric Flow Cytometry

IImmediately upon sacrifice, single-cell suspensions were prepared from tumors. In brief, tumor tissues were minced, and then digested with 3.0 mg/ml collagenase A (Roche) and 50 U/ml DNase I (Sigma-Aldrich) in serum free media for 30 min at 37°C. Cells were filtered through 70μm nylon strainers (Thermo Fisher Scientific) and washed twice in PBS with 2% FBS. Red blood cells were then removed with red blood cell lysis buffer (Sigma-Aldrich). Cells were washed once, blocked with anti-mouse CD16/CD32 blocker and stained in PBS with 0.5% BSA, 2mM EDTA, and 0.01% NaN_3_ with the anti-mouse antibodies. Acquisition was performed on a BD LSRFortessa X-20 Cell Analyzer and the dedicated software Diva (BD). Data were analyzed with FlowJo 10.9.0 software (Tree Star).

**Table:** 

Reagent (mouse)	Fluorophore	Source	Clone	Dilution
CD3e	FITC	Biolegend	17A2	1:400
CD4	APC	BD pharmingen	RM4–5	1:200
CD8a	BUV395	BD Horizon	53 – 6.7	1:200
CD11b	BUV395	BD Biosciences	M1/70	1:400
CD16/32	blocker	Biolegend	93	1:500
CD45	APC Cy7	Biolegend	30-F11	1:400
CD45	BV605	Biolegend	30-F11	1:400
F4/80	BV711	Biolegend	BM8	1:100
Ly6C	APC	Biolegend	HK1.4	1:400
Ly6G	BV421	BD Biosciences	1A8	1:400
NK1.1	BV711	Biolegend	PK136	1:200
Ter119	BV605	Biolegend	TER-119	1:200
CD90.1 (Thy1.1)	eFlour450	ThermoFisher	HIS51	1:200
Fixable viability dye	eFlour780	ThermoFisher	-	1:1000
Zombie UV fixable dye	Indo-1	Biolegend	-	1:200
Reagent (human)	Fluorophore	Source	Clone	Dilution
CD3	ECD	Immunotech	UCHT1	1:50
CD16	PerCP Cy5.5	BD Pharmingen	3G8	1:200
CD45	BV605	Biolegend	2D1	1:20
CD56	PE Cy7	Immunotech	N901	1:100
CD58	PerCP Cy5.5	Biolegend	TS2/9	1:50
B7-H6	PE	R&D systems	875001	1:20
GzmB	AF700	BD Pharmingen	GB11	1:50
HLA-E	APC	Biolegend	3D12	1:20
NKG2D	APC	Invitrogen	1D11	1:20
NKp30	BV785	Biolegend	P30–15	1:20
NKp46	BV421	BD Horizon	9E2	1:20
PRF1	PE	Biolegend	dG9	1:50

### NK cell killing assays

Cytotoxicity of NK cells was assessed in a standard 4-hours flow cytometry-based ex vivo killing assay as previously described^51^. 4×10^4^ tumor cells are plated in 96 well plate and different numbers of NK cells were added one hour later. Recombinant mouse DKK1 (Biolegend) or human DKK1 (Biolegend) was added to a final concentration of 200ng/mL in RPMI1640 media. For each effector: target (E:T) ratio, Percent Specific Killing was calculated as [% 7-AAD^+^ of CTV^+^ cells](Effector +Target) well - [% 7-AAD^+^ of CTV^+^ cel Is] (Targets only) well.

Specific killing of control and rDKK1-stimulated NK cells was also evaluated using IncuCyte Live-cell Analysis system (Satorius). A total of 5×10^3^ mApple^+^ tumor cells were incubated for 2 hours in a 96-well plate and imaged prior to the addition of the NK cells at a 2:1 E:T ratio. Real-time images were captured every 4 hours and up to 48 hours and analyzed using the Incucyte software. Data are presented as red object counts (mApple^+^ cells).

### Immunofluorescence and Confocal microscopy of tumonNK co-cultures

Tumor cells were plated into the poly-D-lysine (Millipore sigma) overnight coated slides and incubated for an hour before adding NK cells. After co-culturing for 3 hours, cells were fixed with 4% PFA and permeabilized with 0.3% Triton X-100. Then stained with F-actin antibody conjugated with Alexa 488 (Invitrogen, A12379) for an hour and mounted with anti-fade fluorescence mounting medium (Abcam). Confocal data was generated on a Nikon AX-R Confocal Microscope (100X objective lens, oil immersion, Numerical Aperture: 1.45, Refractive Index: 1.515) which was purchased with support from the Office of Research Infrastructure Programs (ORIP), a part of the NIH Office of the Director under grant OD030233. Camera setting was set in multi-channel detector mode (FITC and mCherry), Galvano unidirectional scanner with Band scan mode, 4x line averaging, 1.0 μsec dwell time. Images were analyzed with NIS-Elements software (Nikon, ver.5.21.00) and ImageJ^52^.

### Statistical analysis

*In vitro* experiments include technical and biological triplicates and were performed at least 3 times. *In vivo* experiments were done with at least 4 to 9 mice per group (the number of mice used for each experiment is specified in the figure legends) and at least 3 independent experiments were performed. Numerical data are shown as mean +/− SEM. T-test statistical analysis was used to compare two groups (unpaired t-test when comparing murine data sets and paired when comparing human cells from a single donors or patients). In calculating two-tailed significance levels for equality of means, equal variances were assumed for the two populations. In some experiments with multiple groups or time points, analysis of variance (ANOVA), including the Dunnett’s multiple-comparison test, or the Bonferroni’s multiple-comparison test, was used. Results were considered significant at p < 0.05. All statistical analyses were performed with GraphPad Prism 10.2.1 software for Windows (GraphPad Software).

## Figures and Tables

**Table 1.** Characteristics of breast cancer patients including date of sample collection, skeletal and visceral metastasis status, and treatments received at indicated dates (All patients were treated with Denosumab 120mg SQ Q4–12 weeks as an anti-resorptive therapy)

ID	Date of Collection	Bone Met Status	Bone Metastasis	Visceral Metastasis	Treatments and dates (month/year)	
**445**	12/15/2014 06/22/2015	Stable Stable	Sternum, Sacrum, Ilium, Lumbar Spine No changes	Nodal disease Increase R axillary nodes	Letrozole	12/2014 – 06/2015
**550**	09/13/2015 12/07/2017	Stable Stable	Spine, Sternum, Clavicle, Pelvis No Changes	None None	Anastrozole	09/2013 – present
**557**	09/10/2015 01/05/2017	Stable Stable	Spine No changes	Lung No changes	1. Fulvestrant 2. Zoladex	01/2014 - present 01/2014 – 12/2017
**570**	12/10/2015 06/08/2017	Stable Stable	Sternum, Ilium No changes	None None	Anastrozole	05/2014 – present
**571**	09/03/2015 08/11/2016	Stable Stable	Sternum No changes	Lung, Liver, Lymph node Progressive Liver mets	1. Everolimus + Exemestane 2. Capecitabine	03/2015 – 07/2016 07/2016 – 10/2016
**613**	01/10/2017 10/03/2018	Stable Stable	Glenoid No changes	None None	Fulvestrant	09/2016 – 11/2018
**626**	04/08/2017 03/01/2018	Stable Stable	Sternum, Pelvis No changes	None None	Letrozole	12/2016 – 09/2018
**438**	03/30/2015 09/12/2016	Progressive Progressive	Spine, Sternum, Clavicle, Pelvis, Scapula Progression of mets in axial skeleton	Lymph node, Pleural mass, soft tissue Progessive hepatic mets	1. Tamoxifen 2. Letrozole + Palbociclib	10/2013 – 03/2015 06/2015 – 10/2016
**509**	12/02/2015 03/19/2018	Progressive Progressive	Spine, Sacrum Increase T4-T6 Spine Mets	Lung Progessive Lung nodules	Letrozole + Palbociclib	12/2015 – 03/2018
**543**	12/14/2015 10/24/2016	Progressive Progressive	Spine New humerus met	None None	Anastrozole	04/2014 – 03/2016
**564**	10/08/2015 06/23/2016	Progressive Progressive	Ribs, Spine, Sternum Progression of osseous mets	Liver Increase Liver mets	1. Everolimus + Exemestane 2. Tamoxifen	08/2015 – 11/2015 11/2015 – 06/2016
**572**	09/17/2015 03/31/2016	Progressive Progressive	Spine, Ribs, Ilium, Sacrum Progessive mets, New Spine, Femur, Ischium mets	None None	Aromasin	03/2015 – 03/2016
**581**	10/05/2015 08/08/2016	Progressive Progressive	Vertebra, Pelvis New Humerus met	Lymph node, Liver Progressive nodal met	1. Fulvestrant 2. Letrozole + Palbociclib	06/2015 – 01/2016 01/2016 – 06/2016
**611**	11/24/2016 06/21/2017	Progressive Progressive	Diffuse multifocal osseous mets Progression of multifocal osseous mets	None None	Palbociclib + Tamoxifen	05/2016 – 06/2017
**638**	08/22/2017 06/12/2018	Progressive Progressive	Diffuse multifocal osseous mets Progression of multifocal osseous mets	Lung, Liver Progression of Lung/Liver mets	Tamoxifen + AKT inhibitor	07/2017 – 07–2018

Abbreviations: Met, metastasis; SQ, subcutaneous administration; Q4–12 weeks, every 4–12 weeks.
